# How Healthy Lifestyle Habits Have Interacted with SARS-CoV-2 Infection and the Effectiveness of COVID-19 Vaccinations: Tohoku Medical Megabank Project Birth and Three-Generation Cohort Study

**DOI:** 10.31662/jmaj.2024-0043

**Published:** 2024-07-03

**Authors:** Masatsugu Orui, Taku Obara, Mami Ishikuro, Aoi Noda, Genki Shinoda, Keiko Murakami, Tomohiro Nakamura, Hirohito Metoki, Soichi Ogishima, Yoko Izumi, Naoki Nakaya, Atsushi Hozawa, Tadashi Ishii, Fuji Nagami, Masayuki Yamamoto, Shinichi Kuriyama

**Affiliations:** 1 Tohoku Medical Megabank Organization, Tohoku University, Sendai, Japan; 2 Graduate School of Medicine, Tohoku University, Sendai, Japan; 3 Tohoku University Hospital, Tohoku University, Sendai, Japan; 4 Faculty of Data Science, Kyoto Women’s University, Kyoto, Japan; 5 Division of Public Health, Hygiene and Epidemiology, Tohoku Medical and Pharmaceutical University, Sendai, Japan; 6 Advanced Research Center for Innovations in Next-Generation Medicine, Tohoku University, Sendai, Japan; 7 International Research Institute of Disaster Science, Tohoku University, Sendai, Japan

**Keywords:** COVID-19, SARS-CoV-2, vaccine, infection, lifestyle habit, cohort study, booster shot

## Abstract

**Introduction:**

To examine the interaction between lifestyle habits and the COVID-19 vaccinations for preventing SARS-CoV-2 infection, we analyzed 11,016 adult participants registered in the Tohoku Medical Megabank Project Birth and Three-Generation Cohort Study.

**Methods:**

Lifestyle variables, including regular exercise, smoking and drinking habits, sleep status, body mass index, and daily breakfast consumption, were assessed from 2014 to 2019 using baseline questionnaires. Information on SARS-CoV-2 infection and the COVID-19 vaccination were also collected from March 2020 to May 2023. The study period was divided into two in the postvaccination phase: the first period (the beginning of the vaccination program) and the second period (the fourth shot onward).

**Results:**

In the Cox proportional-hazards model analysis, the five-time vaccinations group showed a significantly lower risk of SARS-CoV-2 infection adjusted age, sex, underlying health condition, and lifestyle variables (hazard ratio [HR] 0.81, 95% confidence interval [CI] 0.76-0.86). Logistic regression analysis revealed that a higher number of vaccinations was significantly associated with a low risk of SARS-CoV-2 infection regardless of lifestyle habits (three times in the first period: odds ratio [OR] 0.19, 95% CI 0.15-0.24; five times in the second period: OR 0.07, 95% CI 0.05-0.11 vs. none). Regarding lifestyle habits, the risk reduction in those who had sleep satisfaction (OR 0.12, 95% CI 0.08-0.18) was slightly larger than in those who had sleep dissatisfaction (OR 0.23, 95% CI 0.17-0.32) in the group with the highest number of vaccinations in the first period; however, this interaction was hardly confirmed in the second period when the number of infected cases significantly increased.

**Conclusions:**

Our findings indicated that a higher number of COVID-19 vaccinations was associated with reduced risk of SARS-CoV-2 infection; otherwise, we may need to understand the advantages and limitations of a healthy lifestyle for preventing infection depending on the situation with vaccinations and infection spreading.

## Introduction

The 2019 novel coronavirus disease (COVID-19) pandemic has caused global health and socioeconomic crises due to the lockdown. During this time, numerous studies on lifestyle changes have been conducted, particularly in Japan ^(1), (2), (3), (4), (5), (6), (7), (8), (9)^. However, to the best of our knowledge, there are limited observational studies on how healthy lifestyle habits influence SARS-CoV-2 (severe acute respiratory syndrome coronavirus 2) infection ^(10)^.

Evidently, the COVID-19 vaccine has shown significant advantages for the prevention of SARS-CoV-2 infection and aggravation ^(11), (12), (13)^. In Japan, the Ministry of Health, Labour and Welfare (MHLW) has implemented a vaccination program for COVID-19: (1) The primary vaccination series (two shots) is aimed at reducing COVID-19-related illnesses and severe aggravation. The third shot is a booster shot to enhance the effectiveness of the vaccination. The MHLW has recommended that all adults receive a primary vaccination series and an additional booster shot. (2) The fourth and fifth shots are targeted for the elderly, those who have underlying health conditions, and the healthcare professionals. The completion rate of the primary vaccination series is 79.8% (65 years old and above: 92.7%), and that of the third shot is 67.4% (65 years old and above: 91.7%) ^(14)^. Some studies have assessed the effectiveness of the COVID-19 vaccine against SARS-CoV-2 infection among the general population ^(15), (16), (17), (18)^. At present, no study has evaluated a five-time shot.

Due to a positive association between a healthier lifestyle and improved effect of the COVID-19 vaccine ^(19)^, healthy lifestyle habits, and multiple vaccinations are expected to help prevent SARS-CoV-2 infection; therefore, the interaction between lifestyle habits and multiple shots of the COVID-19 vaccines are worth examining for the prevention of SARS-CoV-2 infection. However, there are limited studies on the aforementioned topic, and these studies only assessed primary vaccination, not the multiple booster shots ^(19), (20)^. Thus, the present study aimed to examine (1) risk reduction associated with COVID-19 vaccination programs against SARS-CoV-2 infections, including a five-time shot, and (2) the interaction between lifestyle habits and the number of COVID-19 vaccinations for the prevention of SARS-CoV-2 infection using data collected from the Tohoku Medical Megabank (TMM) Project Birth and Three-Generation (BirThree) Cohort Study. We hypothesized that (1) the higher the number of COVID-19 vaccinations, the lower the risk of SARS-CoV-2 infection and that (2) there is an interaction between healthier lifestyle habits and higher number of COVID-19 vaccinations, leading to a significantly reduced risk of infection.

## Materials and Methods

### 1. Study design, subject, and record linkage

The TMM aimed to assist medical and health services in overcoming the damages caused by the 2011 Great East Japan Earthquake by supporting survivors and implementing personalized healthcare ^(21), (22)^. The BirThree Cohort Study, which is part of the TMM, recruited participants, including pregnant women and their children after birth, elder children, partners, and parents, in the Miyagi Prefecture and part of the Iwate Prefecture in Japan from July 2013 to March 2017. Detailed information on the recruitment methods is shown in the articles referenced as No. 21 and 22. The cohort participants’ questionnaire data are linked with several public data.

As part of record linkages to cohort data, information of residents with SARS-CoV-2 infection who received the COVID-19 vaccine was also collected after the COVID-19 outbreak. Under the Infectious Diseases Control Law, there is a regulation stipulating that every medical institute must report the information of individuals who tested positive in SARS-CoV-2 Polymerase Chain Reaction (PCR) and antigenic tests to the public health center. Information on SARS-CoV-2 infection is collected by prefectural offices and cities designated by the Japanese government. Starting in September 26, 2022, this reporting method has been simplified: individuals who test positive in the antigen test need to register online. Moreover, under the Immunization Act, basic municipalities are responsible for implementing the vaccine program based on the Act, including in the COVID-19 vaccines. The Sendai City office can collect SARS-CoV-2 infection and COVID-19 vaccination information; therefore, Sendai City was set as the subject area of this follow-up study.

**Figure 1.**
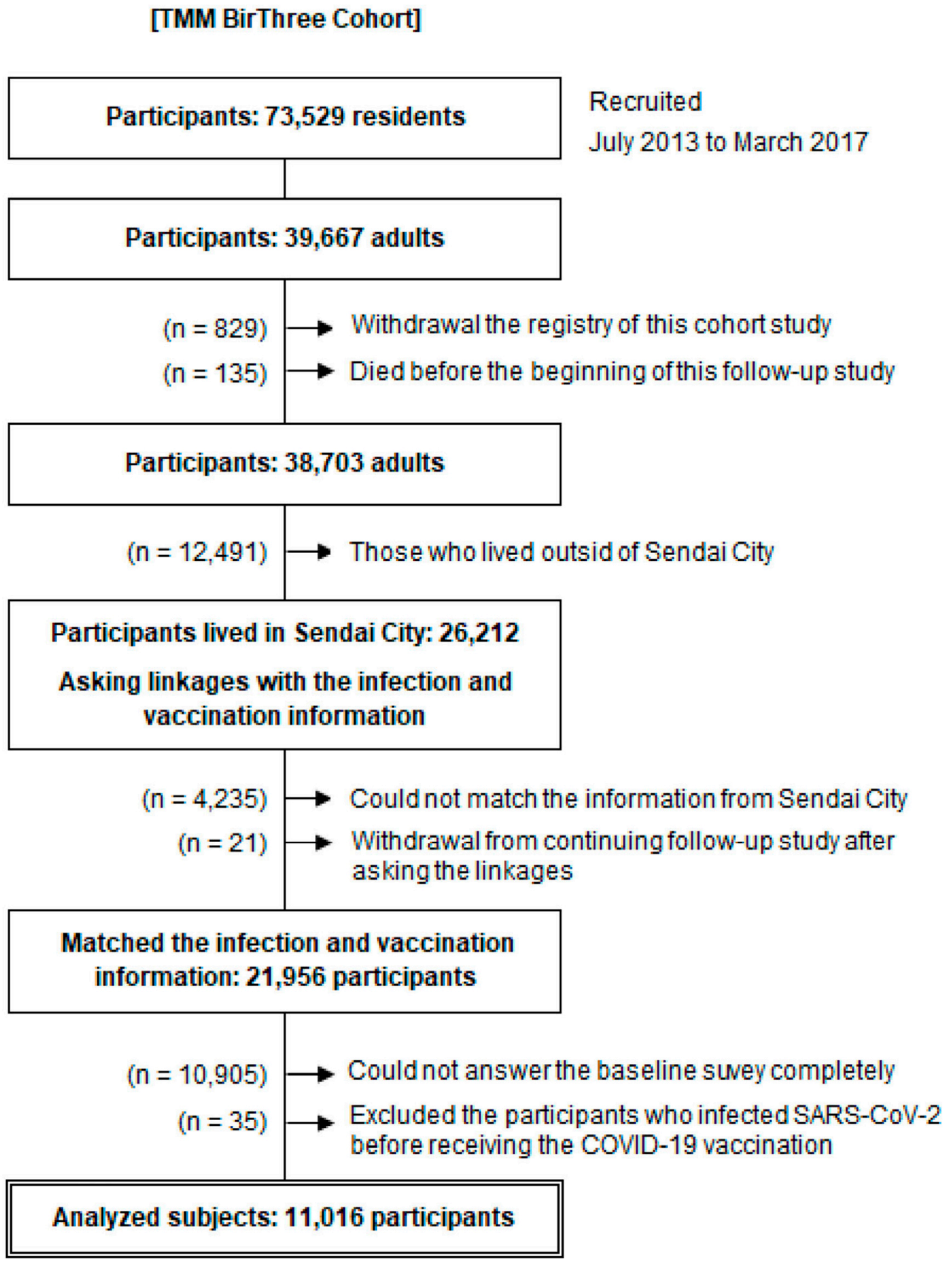
Participants of the TMM BirThree Cohort Study cohort and analyzed subjects The TMM BirThree Cohort Study recruited 73,529 participants from July 2013 to March 2017, of whom 39,667 were adults. Among the adult participants, 26,212 who were residing in Sendai City were linked to the information on the COVID-19 vaccination and SARS-CoV-2 infection. Finally, 11,016 adult participants who completed the questionnaire were analyzed.

Of the 73,529 participants, 39,667 were adults. Among them, 829 withdrew from the study and 135 died before the start of the study. Additional 12,491 participants who lived outside of Sendai City were excluded. We asked the Sendai City Office to match the infection and vaccination information with 26,212 participants living in Sendai City. Of the participants, 4,235 did not match the information collected from Sendai City due to relocation to other municipalities or other reasons. During the matching, a total of 21 participants withdrew from the study. We excluded 10,905 participants who could not completely answer the baseline survey and 35 participants due to infection with SARS-CoV-2 before receiving the COVID-19 vaccine. Finally, 11,016 adult participants were analyzed (Figure 1). The participants answered the baseline questionnaire regarding lifestyle habits and past medical history during the baseline survey.

### 2. Lifestyle habits

We assessed the participants’ lifestyle variables, including regular exercise, smoking and drinking habits, sleep status, body mass index (BMI), and daily breakfast consumption, during the baseline survey using Breslow’s healthy lifestyle as a reference ^(23)^.

The participants answered the following questions regarding regular exercise habits: (1) the type of exercise and activity: “brisk walking,” “light-to-moderate-intensity exercise and activity (e.g., golf, gateball, and gardening/crop work),” and “vigorous exercise (e.g., tennis, jogging, aerobics, and swimming)”; (2) the frequency of exercise and activity: “none,” “less than once per month,” “once to three times per month,” “once to twice per week,” “three to four times per week,” and “almost every day”; and (3) the duration of the exercise and activity: “less than 30 min,” “30 min to 1 h,” “1 to 2 h,” “2 to 3 h,” and “more than 4 h.” When combing the answers regarding the frequency and duration, we defined regular exercise habits as corresponding to the amount of exercise: “more than 30 min in an exercise and activity, at least twice per week or more” based on the national health promotion measures “Health Japan 21” ^(24), (25)^.

Smoking habit was assessed based on the current smoking status. “Nonsmokers” were those who had no experience smoking, “past smokers” were those who had quit smoking, and “current smokers” were those who have continued smoking regardless of the daily cigarette consumption.

As regards drinking habit, the participants were asked the following questions: “Do you drink alcohol more than once a month?” and “If you are a drinker, what kind of alcohol and how much do you drink on a typical drinking day?” Then, we defined those who drink alcohol as “moderate drinkers” or “heavy drinkers” based on the amount of alcohol consumed on a typical drinking day. The cutoff points for moderate and heavy drinking were set at two and six drinks per day, respectively ^(24), (25)^. Two drinks were defined as 60 mL of whiskey or brandy, 240 mL of wine, 500 mL of beer, or 180 mL of Japanese sake. Subsequently, we categorized the drinking behaviors into four groups: “nondrinkers,” “moderate drinkers,” “more than moderate to heavy drinkers,” and “heavy drinkers.”

Sleep status was assessed on a four-point scale ranging from “satisfied,” “a little dissatisfied,” and “quite dissatisfied” to “really dissatisfied or cannot sleep at all.” As an original classification, we divided the responses into two categories, namely, “satisfied” and “dissatisfied,” with “a little dissatisfied” as the boundary.

For BMI, we categorized individuals into four groups as follows: (1) less than 18.5 kg/m^2^ as underweight, (2) 18.5 kg/m^2^ and more to less than 25.0 kg/m^2^ as having a standard weight, (3) 25.0 kg/m^2^ and more to less than 30.0 kg/m^2^ as overweight, and (4) 30 kg/m^2^ and more as obese ^(26)^.

Breakfast consumption, which served as an indicator of daily dietary habits, was assessed by asking the participants “How often do you eat breakfast?” on a six-point scale ranging from “less than once a month,” “one to three times a month,” “one to two times a week,” “three to four times a week,” “five to six times a week,” and “every day.” We then categorized the responses into two groups: “having every day” and “others.”

### 3. Underlying health condition

Individuals with underlying health conditions were defined as those having a past medical history of any cancer, chronic heart disease, hypertension, cerebral stroke, chronic obstructive pulmonary disease, chronic kidney disease including renal dialysis, and diabetes mellitus ^(19), (27)^.

### 4. SARS-CoV-2 infection and COVID-19 vaccination

#### 4-1. Definition of the infection and follow-up period

In this study, the beginning of the follow-up period was March 1, 2020, as the first SARS-CoV-2 infection case in Sendai City was confirmed during this time. We defined SARS-CoV-2 infection as positivity in the SARS-CoV-2 PCR and antigenic test which the test result was registered to regional public health center until May 7, 2023 ^(28)^.

#### 4-2. Study period according to the vaccination program

**Figure 2.**
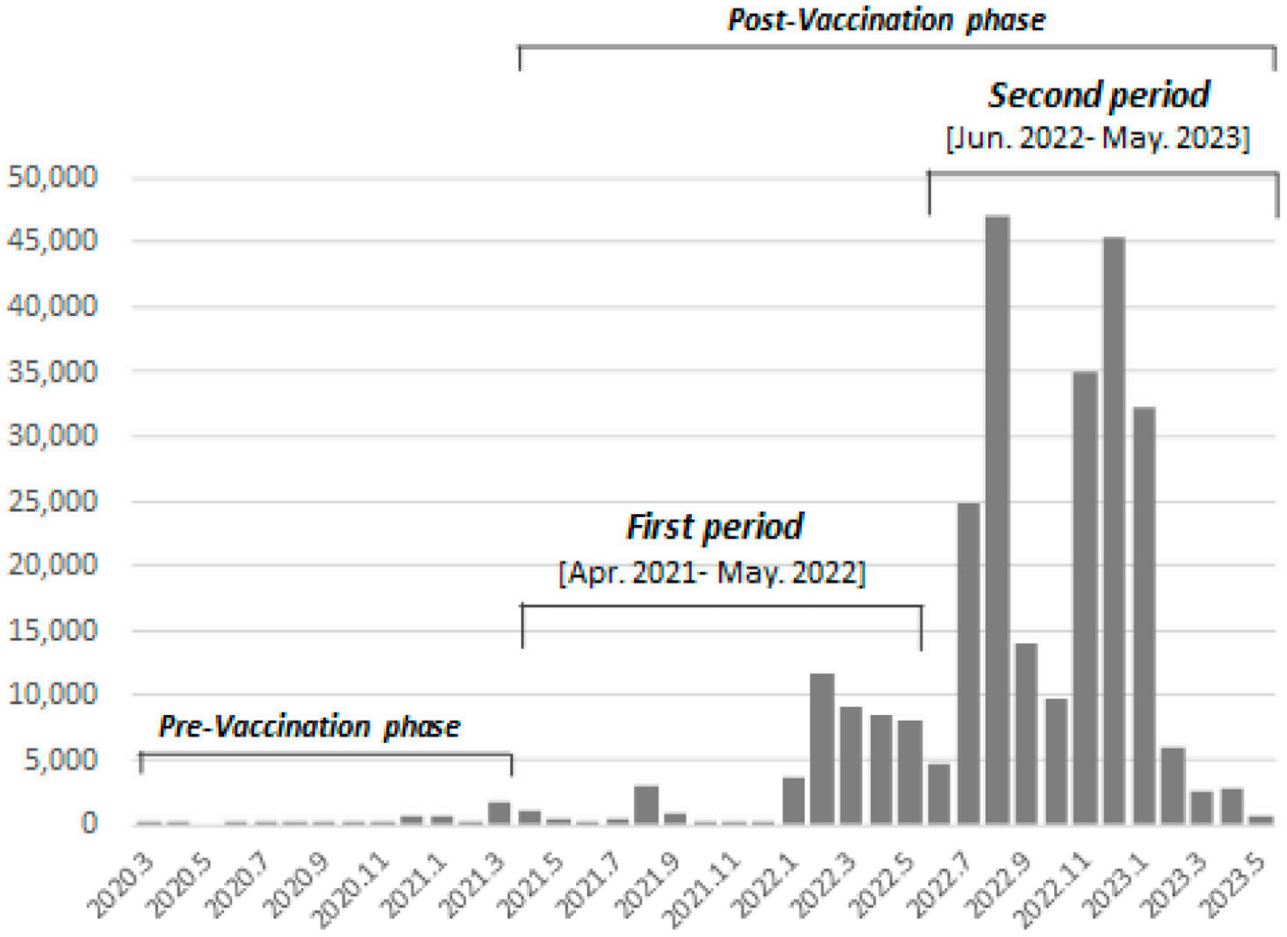
Number of SARS-CoV-2 cases and definition of the two periods The first period was from April 2021 to May 2022, which was the beginning phase of the COVID-19 vaccination program targeting the entire adult population. The second period was from June 2022 onward, which was initiated with a fourth shot targeting the elderly, those who have underlying health conditions, and healthcare professionals.

We divided the study period in the postvaccination phase into two as we focused on the difference in the number of vaccinations and the target population: (1) the first period, from April 2021 to May 2022, which was the beginning phase of the COVID-19 vaccination program (the primary vaccination series and the first booster shot) targeting whole adults, and (2) the second period, from June 2022 onward, which was initiated with a fourth shot targeting the elderly, those who have underlying health conditions, and healthcare professionals. The number of infected cases rapidly increased from the middle of the first period and then exploded in the second period (Figure 2).

#### 4-3. Group of the number of vaccinations

In this study, the participants who had not received a vaccine were assigned to the “none” group. Conversely, those who received the vaccine (BNT162b2, mRNA-1273, and NVX-Cov2373) once were assigned to the “once” group and those who received the vaccine twice (twice with the same type of vaccine or in combination with BNT162b2, mRNA-1273, and NVXCoV-2373) assigned to the “twice group.” Furthermore, participants who have received the vaccine more than three times were assigned to the “three-time,” “four-time,” and “five-time” groups regardless of the type of vaccines. If the participant was infected between the vaccine series, we did not count the vaccination numbers after the infection.

### 5. Data analysis

First, we performed a simple tabulation of the participants’ basic characteristics, lifestyle habits, and vaccination times. Then, we conducted Kaplan-Meier estimate and Cox proportional-hazards model analysis while adjusting for age, sex, underlying health conditions, and lifestyle habits. The same subject was not included more than once and was classified based on the final number of vaccinations. The index date was set as the final date of receiving the COVID-19 vaccination. Finally, we conducted logistic regression analyses to examine the interaction between SARS-CoV-2 infection and the number of COVID-19 vaccinations. We stratified lifestyle habits to explore how healthy lifestyle habits influence risk reduction associated with vaccination. The logistic regression analyses were conducted while adjusting for age, sex, and underlying health conditions.

The first period in this study corresponds to the primary vaccination series and the third shot was implemented, therefore information on four- and five-time vaccinations was not included. The participants with SARS-CoV-2 infection during the first period were excluded from the analysis in the second period. In addition, we analyzed the participants who were infected with SARS-CoV-2 at least 7 days after the final vaccination referring to a previous review study ^(11)^. A total of 65 participants were found to have been infected with SARS-Cov-2 twice; thus, we conducted analysis based on the first infection record.

Statistical significance was evaluated using two-sided, design-based tests with a 5% significance level using Stata 15 (StataCorp, 2017; Stata Statistical Software: Release 15; College Station, TX: StataCorp LLC).

### 6. Ethical considerations

This study was approved by the Ethical Research Committee of Tohoku University Graduate School of Medicine (2013-4-103, approval date: May 10, 2013; latest revision 2023-4-040, approval date: June 21, 2023). We obtained informed consent including record linkages to cohort data from all participants in the TMM BirThree Cohort Study.

## Results

### 1. Basic characteristics and lifestyle habits

The 30-39-year age group had the highest proportion of participants because pregnant women were recruited initially, and later, their partners or parents were recruited to the BirThree Cohort Study. For the number of vaccinations, the three-time group had the largest proportion in each category during the first and second periods (60.5% and 36.1%, respectively). Lifestyle variables, such as regular exercise, smoking and drinking habits, sleep status, body weight, and breakfast consumption, did not exhibit notable trends (Table 1).

**Table 1.** Basic Characteristics and Number of Vaccinations in the Two Periods.

		Total		1st period [2021.4-2022.5]				2nd period [2022.6-2023.5]			
				Positive		Negative		Positive		Negative	
		n = 11,016		n = 808		n = 10,208		n = 2,202		n = 8,006	
		n	%	n	%	n	%	n	%	n	%
Age group	20-29	469	4.3	42	5.2	427	4.2	128	5.8	299	3.7
	30-39	5,235	47.5	486	60.1	4,749	46.5	1,239	56.3	3,510	43.8
	40-49	2,892	26.3	203	25.1	2,689	26.3	577	26.2	2,112	26.4
	50-59	441	4.0	29	3.6	412	4.0	57	2.6	355	4.4
	60-69	1,304	11.8	37	4.6	1,267	12.4	143	6.5	1,124	14.0
	70-79	641	5.8	10	1.2	631	6.2	57	2.6	574	7.2
	80-89	34	0.3	1	0.1	33	0.3	1	0.0	32	0.4
Sex	Women	7,337	66.9	567	70.4	6,770	66.6	1,506	68.8	5,264	66.0
Underlying health condition	Having	1,211	11.0	47	5.8	1,164	11.5	178	8.1	986	12.4
Regular exercise	Having	2,832	25.9	160	20.0	2,672	26.4	516	23.6	2,156	27.1
Smoking habit	Non	6,528	60.0	461	57.6	6,067	60.2	1,325	60.8	4,742	60.0
	Past	2,916	26.8	234	29.3	2,682	26.6	585	26.9	2,097	26.5
	Current	1,437	13.2	105	13.1	1,332	13.2	268	12.3	1,064	13.5
Drinking habit	Non	5,834	54.1	475	60.1	5,359	53.6	1,220	57.0	4,139	52.7
	Moderate	1,430	13.3	96	12.2	1,334	13.4	259	12.1	1,075	13.7
	More moderate to less heavy	1,560	14.5	90	11.4	1,470	14.7	299	14.0	1,171	14.9
	Heavy	1,955	18.1	129	16.3	1,826	18.3	364	17.0	1,462	18.6
Sleep status	Satisfaction	4,196	38.3	266	33.1	3,930	38.8	790	36.1	3,140	39.5
BMI	Less than 18.5	1,154	10.7	89	11.2	1,065	10.6	236	11.0	829	10.6
	18.5-25.0	7,645	70.8	575	72.5	7,070	70.7	1,533	71.2	5,537	70.5
	25.0-30.0	1,665	15.4	101	12.7	1,564	15.6	322	15.0	1,242	15.8
	30 and more	332	3.1	28	3.5	304	3.0	61	2.8	243	3.1
Breakfast consumption	Every day	7,987	75.2	558	72.7	7,429	75.4	1,563	73.8	5,866	75.8
No. of Vaccination	None	972	8.8	137	17.0	835	8.2	-	-	-	-
[1st period]	Once	40	0.4	8	1.0	32	0.3	-	-	-	-
	Twice	3,333	30.3	476	58.9	2,857	28.0	-	-	-	-
	Three	6,671	60.6	187	23.1	6,484	63.5	-	-	-	-
[2nd period]	None	924	8.4	-	-	-	-	181	8.2	642	8.0
	Once	39	0.4	-	-	-	-	8	0.4	23	0.3
	Twice	2,323	21.1	-	-	-	-	493	22.4	1,339	16.7
	Three times	3,991	36.2	-	-	-	-	1,194	54.2	2,597	32.4
	Four times	1,974	17.9	-	-	-	-	283	12.9	1,685	21.0
	Five times	1,765	16.0	-	-	-	-	43	2.0	1,720	21.5

### 2. Time-dependent analysis of the COVID-19 vaccination and SARS-CoV-2 infection

**Figure 3.**
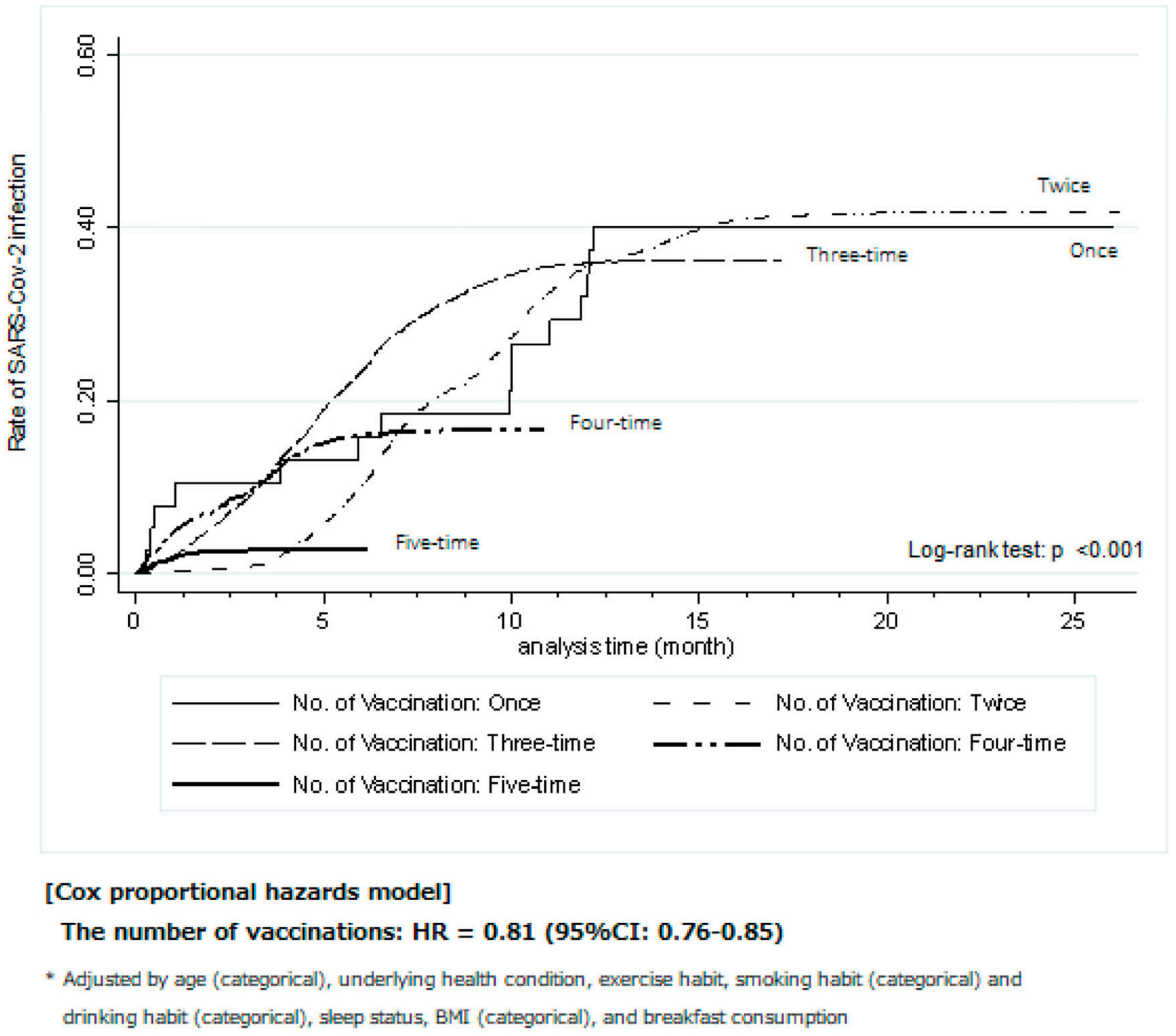
Time-dependent variables of the final date of vaccination and SARS-CoV-2 infection (first and second periods: 2021.4-2023.5) Vertical axis: Rate of SARS-CoV-2 infection. Horizontal axis: Analysis time (months). Cox proportional-hazards model analysis was conducted while adjusting for age (categorical), underlying health conditions, exercise habits, smoking and drinking habits (categorical), sleep status, body mass index (categorical), and breakfast consumption.

The Kaplan-Meier method was employed to illustrate the trends in the rate of SARS-CoV-2 infection for each number of vaccinations from the final date of vaccination, as shown in Figure 3. A higher number of COVID-19 vaccinations was associated with a lower rate of SARS-CoV-2 infection (log-rank test: *P* < 0.001). Specifically, the infection rate of the five-time group was extremely lower than that of the other groups. In the Cox proportional-hazards model analysis, a higher number of vaccinations showed a significantly lower risk of SARS-CoV-2 infection (hazard ratio [HR] 0.81, 95% confidence interval [CI] 0.76-0.86). In the first period alone, the finding was similar to that in the second period (HR 0.75, 95% CI 0.62-0.91; see Supplementary Figure 1).

### 3. Logistic regression analysis of lifestyle habits and the number of vaccinations with SARS-CoV-2 infection

**Figure 4.**
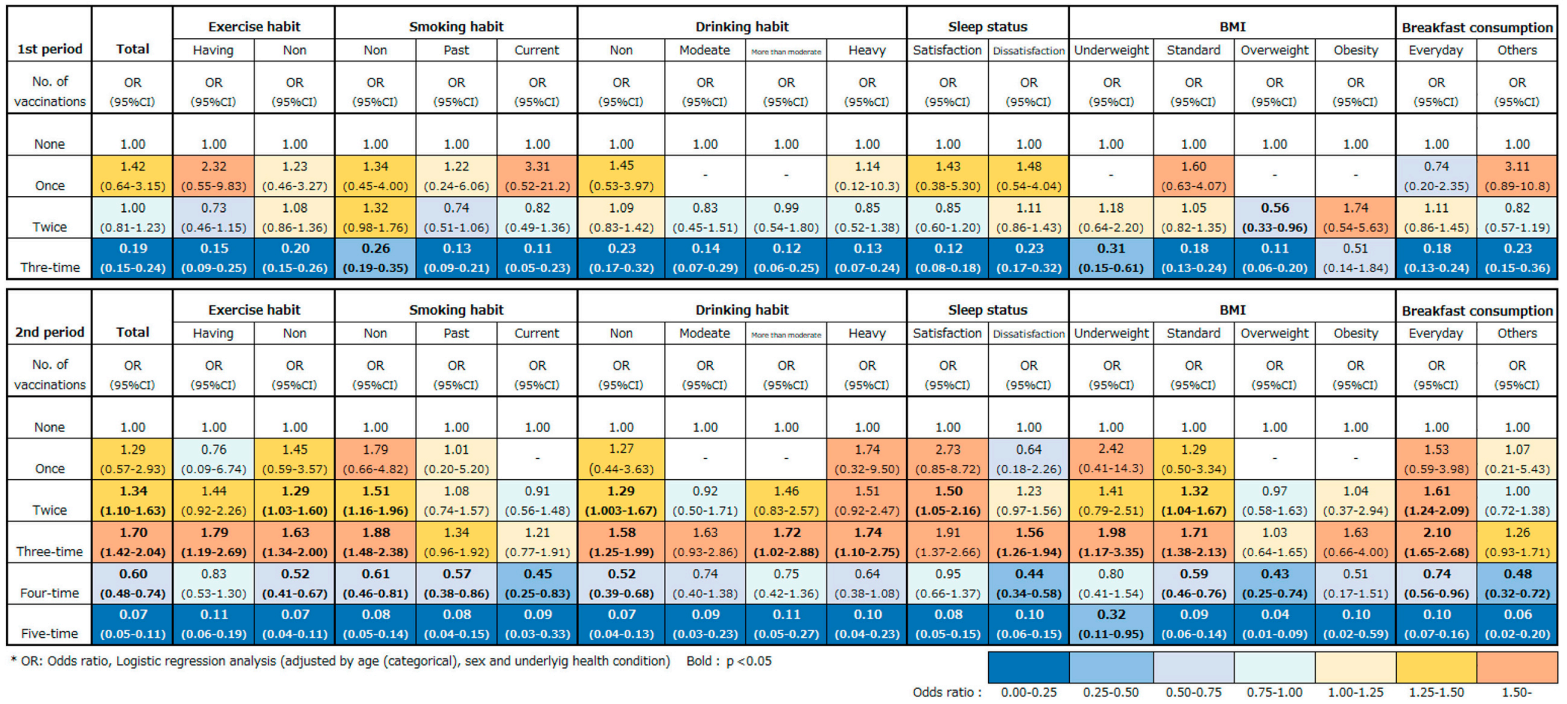
Visualization of interaction between lifestyle habits and the number of vaccinations against SARS-CoV-2 infection Logistic regression analyses were conducted stratifying the lifestyle variables. The figure in the upper portion illustrates the first period, whereas that in the lower portion shows the second period. Blue indicates lower risk of SARS-CoV-2 infection, whereas red indicates higher risk of infection.

When analyzing the interaction of lifestyle habits with SARS-CoV-2 infection and the COVID-19 vaccinations, the “none” vaccination group in each lifestyle habit category was used as reference. The findings of the logistic regression analyses are shown in Figure 4. In the first period, the three-time group had a low risk of SARS-CoV-2 infection regardless of the lifestyle habits (see the “Total” column). Among the participants in the three-time group, those with regular exercise habits, with past and current smoking habits, with moderate and heavy drinking habits, with sleep satisfaction, and who were overweight had a lower risk compared with the total OR and other categories of the same lifestyle variables at the three-time shots.

In the second period, both the four- and five-time groups had a low risk of infection against SARS-CoV-2 regardless of the lifestyle habits. However, the difference in risk reduction between healthy and unhealthy lifestyle habits was negligible compared with that of the first period, except for BMI (Figure 4).

## Discussion

According to the National Vaccination Plan ^(14)^, the BNT162b2 (started in February 2021, limited to healthcare professionals), mRNA-1273 (started in May 2021), and NVX-Cov2373 (started in June 2022) vaccines were gradually administered. The vaccine types varied even within the same period in this study. Nevertheless, in our findings, a higher number of vaccinations showed significant associations with a lower risk of SARS-CoV-2 infection, particularly in the second period. These findings may be related to immunological memory effects ^(29)^ and leading to more protection from reinfection due to booster effects ^(20)^. Therefore, our findings suggest that COVID-19 vaccination programs with multiple booster shots are effective for the prevention of SARS-CoV-2 infection.

The rate of SARS-CoV-2 infection among those who received COVID-19 vaccination once, twice, and thrice was much higher than that of the four- and five-time groups. This could be attributed to the longer duration from the final vaccination date. Because the mean time from the final vaccination among the one-, two-, and three-time groups was much longer (see also Supplementary Table 1), the effectiveness of COVID-19 vaccination is expected to decrease ^(30)^. Because of the longer duration from the final vaccination date, the circulated strains and epidemic scales were changed by the emergence of the Omicron variant. Therefore, the effectiveness of the vaccines was expected to weaken due to immune escape ^(31), (32)^.

It should also be considered that those who received a five-time shot were postponed the index date of receiving the vaccine, in other words, it could shorten the index date from the final vaccination. Additionally, it might have a high level of self-discipline in terms of health control, including lifestyle habits. Those willing to receive vaccines tended to adhere to infection prevention measures such as washing hands and wearing masks ^(33)^. Furthermore, the rates of SARS-CoV-2 infection extremely varied depending on age. The rates among the elderly were much lower than those among the younger generation and middle-aged individuals (see Supplementary Table 2). This may be related to the decreasing frequency of outings ^(34), (35)^, which could be related to strict self-quarantine, and the high number of vaccinations. Thus, the characteristics of the five-time group, especially, the high proportion of the elderly should be noted (see Supplementary Table 1).

As regards the association among healthy lifestyle habits, COVID-19 vaccination, and SARS-CoV-2 infection, a previous review study has reported that several lifestyle habits reduced the risk of SARS-CoV-2 infection (e.g., regular exercise: 15% reduction, extra 1 h of sleep: 12% reduction, and standard weight: 63% reduction) ^(36)^. In our findings, when analyzed with the stratification of lifestyle habits, being overweight had a greater effect on reducing the risk of infection in both periods. Conversely, being underweight had a relatively negligible effect on reducing the risk of SARS-CoV-2 infection. Evidently, obesity is one of the most critical risk factors for SARS-CoV-2 infection ^(37), (38), (39), (40), (41)^, but it was considered obesity (BMI ≤ 30 kg/m^2^), not overweight (25 kg/m^2^ ≤ BMI < 30 kg/m^2^) ^(37), (39), (40), (41)^. In a previous study, evidence suggested a J-shaped nonlinear relationship between BMI and mortality from COVID-19. Even though the difference in the infection risk of those who are underweight and overweight during the prevaccination phase should be considered (Supplementary Figure 2), the effect of BMI involved in underweight and overweight may be essential for considering the interaction between the COVID-19 vaccinations and SARS-CoV-2 infection.

Furthermore, our findings indicated that the risk reduction effects of SARS-CoV-2 infection in those with sleep satisfaction were greater than in those with sleep dissatisfaction, but only in the first period. In the second period, our findings hardly confirmed these interactions. When COVID-19 vaccination was not considered, healthier lifestyle habits showed a lower risk of the infection in the prevaccination phase, but in the postvaccination phase, these associations were hardly confirmed (see Supplementary Figure 2). This could be attributed to the fact that the influence of lifestyle habits on SARS-CoV-2 infection has relatively decreased due to the rapid increase in the number of infected cases (Figure 2). Therefore, it may be necessary to understand both the advantages and limitations of a healthier lifestyle habit for preventing SARS-CoV-2 infection.

This study has several limitations. First, information regarding lifestyle habits and underlying health conditions was obtained at the time of the baseline survey, not before the SARS-CoV-2 infection. Second is the representativeness of these participants. As shown in Table 1, the proportion of individuals in their 30s and women significantly differs compared with the general community in Japan. Third, the rates of SARS-CoV-2 infection extremely varied depending on age, with higher rates observed in younger and middle-aged groups compared with the elderly. Such differences in SARS-CoV-2 infection rates by age group may have influenced our findings. Fourth, we could not collect the strains of SARS-CoV-2. Furthermore, the two periods in this study do not completely correspond to the circulated strains of SARS-CoV-2. Therefore, we were not able to evaluate while considering the difference in the circulation strains of SARS-CoV-2. Finally, the characteristic of the five-time group may be limited. As mentioned in the Discussion section, those who received a higher number of vaccinations might have high self-discipline for infection prevention. Further studies are warranted to control selection bias.

Despite the several limitations, this study also has the following strengths: (1) We could collect official information on SARS-CoV-2 infection cases and COVID-19 vaccination, which are more accurate than self-report-based information. (2) Moreover, it is possible to grasp information on lifestyle habits before the COVID-19 outbreak within a large, community-based population. (3) Furthermore, we could evaluate the interaction between healthy lifestyle habits and the COVID-19 vaccination for the prevention of SARS-CoV-2 infection for an extended duration, exceeding the timeframe of previous studies.

In conclusion, we present the several important findings of this study: (1) The higher the number of COVID-19 vaccinations, the lower the rate of SARS-CoV-2 infection. (2) A higher number of vaccination showed a significant association with a greater reduction of the risk of SARS-CoV-2 infection regardless of the lifestyle habits. However, the effect of lifestyle habits is relatively negligible. We hope that our findings can contribute to future infection control by providing insights into the advantages and limitations of a healthier lifestyle habit for the prevention of infection depending on the situation with vaccinations and infection spreading.

## Article Information

### Conflicts of Interest

None

### Sources of Funding

This work was supported by the Ministry of Education, Culture, Sports, Science and Technology (MEXT), the Japan Agency for Medical Research and Development [JP17km0105001, JP21tm0124005 and JP21tm0424601], and JST SPRING [JPMJSP2114].

### Acknowledgement

TMM BirThree Cohort Study, especially a follow-up survey collecting health information, was conducted, thanks to the collaborative municipalities, including the Sendai City Office. Also, this study was supported by grants from the Ministry of Education, Culture, Sports, Science and Technology (MEXT), the Japan Agency for Medical Research and Development [JP17km0105001, JP21tm0124005 and JP21tm0424601], and JST SPRING [JPMJSP2114]. Our research members are as follows:

https://www.megabank.tohoku.ac.jp/english/a230901/

### Author Contributions

Masatsugu Orui conceptualized and designed this study, conducted the initial analyses, drafted the initial manuscript, and revised the manuscript. Also, managed the implementation of the BirThree Cohort Study.

Prof. Shinichi Kuriyama and Dr. Taku Obara conceptualized, designed, and organized the BirThree Cohort Study and reviewed the manuscript.

Dr. Mami Ishikuro, Dr. Keiko Murakami, Dr. Aoi Noda, and Mr. Genki Shinoda managed the implementation of the BirThree Cohort Study and reviewed the manuscript.

Prof. Masayuki Yamamoto, Prof. Fuji Nagami, Prof. Tadashi Ishii, Prof. Atsushi Hozawa, Prof. Yoko Izumi, Prof. Soichi Ogishima, Prof. Naoki Nakaya, Prof. Hirohito Metoki, and Prof. Tomohiro Nakamura supervised the implementation of the BirThree Cohort Study, supported the data collection of SARS-CoV-2-infected cases and those who received the COVID-19 vaccine, and reviewed the manuscript critically.

All authors approved the final manuscript as submitted and agreed to be accountable for all aspects of the work.

### Approval by Institutional Review Board (IRB)

The Ethical Research Committee at Tohoku University Graduate School of Medicine (2013-4-103, approval date: May 10, 2013; latest revised 2023-4-040, approval date: June 21, 2023).

### Data Availability

All data used to support the findings may be released upon application to the Tohoku Medical Megabank Organization.

## Supplement

Supplementary Figure 1

Supplementary Figure 2

Supplementary Table 1

Supplementary Table 2
