# Supplementary material for: How Healthy Lifestyle Habits Have Interacted with SARS-CoV-2 Infection and the Effectiveness of COVID-19 Vaccinations: Tohoku Medical Megabank Project Birth and Three-Generation Cohort Study
Source: JMA J. 2024 Jul 3;7(3):353–63. doi: 10.31662/jmaj.2024-0043 (PMC11301014; doi:10.31662/jmaj.2024-0043)
Supplement: Supplementary Figure 1 [file 2433-3298-7-3-0353-s001.pdf]

**Supplementary Table 1** Basic characteristics by sort of the number of vaccinations

| | | **Number of vaccinations** [2021.4-2023.5] | | | | | | | | | | | |
|---|---|---|---|---|---|---|---|---|---|---|---|---|---|
| | | **None** | | **Once** | | **Twice** | | **Three-time** | | **Four-time** | | **Five-time** | |
| | | n= 959 | | n= 39 | | n= 2,323 | | n= 3,991 | | n= 1,974 | | n= 1,765 | |
| | | n | % | n | % | n | % | n | % | n | % | n | % |
| **Age** | 20-29 | 106 | 11.1 | 3 | 7.7 | 160 | 6.9 | 150 | 3.8 | 46 | 2.3 | 7 | 0.4 |
| | 30-39 | 540 | 56.3 | 26 | 66.7 | 1,439 | 61.9 | 2,291 | 57.4 | 739 | 37.4 | 215 | 12.2 |
| | 40-49 | 212 | 22.1 | 7 | 17.9 | 620 | 26.7 | 1,231 | 30.8 | 675 | 34.2 | 156 | 8.8 |
| | 50-59 | 37 | 3.9 | 3 | 7.7 | 48 | 2.1 | 120 | 3.0 | 130 | 6.6 | 105 | 5.9 |
| | 60-69 | 44 | 4.6 | 0 | 0.0 | 41 | 1.8 | 150 | 3.8 | 277 | 14.0 | 796 | 45.1 |
| | 70-79 | 19 | 2.0 | 0 | 0.0 | 13 | 0.6 | 46 | 1.2 | 102 | 5.2 | 463 | 26.2 |
| | 80-89 | 1 | 0.1 | 0 | 0.0 | 2 | 0.1 | 3 | 0.1 | 5 | 0.3 | 23 | 1.3 |
| **Sex** | Women | 654 | 68.3 | 29 | 74.4 | 1,541 | 66.8 | 2,684 | 67.5 | 1,314 | 66.8 | 1,141 | 65.0 |
| **Underlying health condition** | Having | 60 | 6.3 | 2 | 5.1 | 106 | 4.6 | 220 | 5.5 | 243 | 12.3 | 586 | 33.2 |
| **Exercise habit** | Having | 191 | 20.1 | 9 | 23.7 | 429 | 18.6 | 871 | 22.0 | 512 | 26.0 | 827 | 47.1 |
| **Smoking habit** | Non | 547 | 57.7 | 23 | 59.0 | 1,264 | 55.2 | 2,382 | 60.3 | 1,218 | 62.3 | 1,111 | 64.1 |
| | Past | 245 | 25.8 | 10 | 25.6 | 650 | 28.4 | 1,042 | 26.4 | 525 | 26.9 | 454 | 26.2 |
| | Current | 156 | 16.5 | 6 | 15.4 | 377 | 16.5 | 526 | 13.3 | 211 | 10.8 | 168 | 9.7 |
| **Drinking habit** | Non | 549 | 59.1 | 23 | 65.7 | 1,326 | 58.3 | 2,184 | 56.0 | 1,016 | 52.5 | 755 | 43.4 |
| | Moderate | 98 | 10.5 | 1 | 2.9 | 279 | 12.3 | 481 | 12.3 | 270 | 14.0 | 303 | 17.4 |
| | Moderate to heavy | 117 | 12.6 | 3 | 8.6 | 272 | 12.0 | 542 | 13.9 | 287 | 14.8 | 343 | 19.7 |
| | Heavy | 165 | 17.8 | 8 | 22.9 | 398 | 17.5 | 691 | 17.7 | 362 | 18.7 | 340 | 19.5 |
| **Sleep status** | Satisfaction | 308 | 32.5 | 16 | 41.0 | 780 | 33.8 | 1,436 | 36.3 | 769 | 39.0 | 897 | 51.1 |
| **BMI** | Less than 18.5 | 126 | 13.4 | 6 | 16.2 | 271 | 12.0 | 475 | 12.2 | 174 | 8.9 | 105 | 6.0 |
| | More than 18.5 to 25.0 | 645 | 68.8 | 29 | 78.4 | 1,615 | 71.6 | 2,764 | 70.9 | 1,391 | 71.1 | 1,221 | 70.1 |
| | More than 25.0 to 30.0 | 128 | 13.7 | 2 | 5.4 | 306 | 13.6 | 557 | 14.3 | 325 | 16.6 | 359 | 20.6 |
| | 30 and more | 38 | 4.1 | 0 | 0.0 | 65 | 2.9 | 104 | 2.7 | 67 | 3.4 | 58 | 3.3 |
| **Breakfast consumption** | Everyday | 576 | 63.2 | 26 | 68.4 | 1,509 | 67.9 | 2,828 | 73.6 | 1,537 | 80.3 | 1,533 | 88.6 |
| **Time from the last vaccination** | Month (mean/SD) | - | - | 15.0 | 7.5 | 14.7 | 5.8 | 9.6 | 4.3 | 5.4 | 2.1 | 4.7 | 1.0 |
