# Supplementary material for: How Healthy Lifestyle Habits Have Interacted with SARS-CoV-2 Infection and the Effectiveness of COVID-19 Vaccinations: Tohoku Medical Megabank Project Birth and Three-Generation Cohort Study
Source: JMA J. 2024 Jul 3;7(3):353–63. doi: 10.31662/jmaj.2024-0043 (PMC11301014; doi:10.31662/jmaj.2024-0043)
Supplement: Supplementary Figure 2 [file 2433-3298-7-3-0353-s002.pdf]

Supplementary Table 2 SARS-CoV-2 infection rates for each age group and the number of vaccinations

			SARS-Cov-2: Positive			
			Positive Rates		(Hospitalization)	
			n	%	n	%
**1st period [2021.4-2022.5]***						
Overall		(n=11,016)	808	7.3	10	0.1
Age	20-29	(n=469)	42	9.0	1	0.2
	30-39	(n=5,235)	486	9.3	5	0.1
	40-49	(n=2,892)	203	7.0	1	0.0
	50-59	(n=441)	29	6.6	1	0.2
	60-69	(n=1,304)	37	2.8	1	0.1
	70-79	(n=641)	10	1.6	1	0.2
	80-89	(n=34)	1	2.9	0	0.0
No. of vaccination	None	(n=972)	137	14.1	2	0.2
	Once	(n=40)	8	20.0	0	0.0
	Twice	(n=3,333)	476	14.3	8	0.2
	Three times	(n=6,671)	187	2.8	0	0.0
**2nd period [2022.6-2023.5]***						
Overall		(n=10,208)	2,202	21.6	16	0.2
Age	20-29	(n=427)	128	30.0	1	0.2
	30-39	(n=4,749)	1,239	26.1	6	0.1
	40-49	(n=2,689)	577	21.5	5	0.2
	50-59	(n=412)	57	13.8	1	0.2
	60-69	(n=1,267)	143	11.3	2	0.2
	70-79	(n=631)	57	9.0	1	0.2
	80-89	(n=33)	1	3.0	0	0.0
No. of vaccination	None	(n=823)	181	22.0	2	0.2
	Once	(n=31)	8	25.8	0	0.0
	Twice	(n=1,832)	493	26.9	2	0.1
	Three times	(n=3,791)	1,194	31.5	6	0.2
	Four times	(n=1,968)	283	14.4	5	0.3
	Five times	(n=1,763)	43	2.4	1	0.1

* Excluded participants who have infected SARS-Cov-2 in the previous period.

* 65 participants were infected SARS-Cov-2 twice.
