# Supplementary material for: How Healthy Lifestyle Habits Have Interacted with SARS-CoV-2 Infection and the Effectiveness of COVID-19 Vaccinations: Tohoku Medical Megabank Project Birth and Three-Generation Cohort Study
Source: JMA J. 2024 Jul 3;7(3):353–63. doi: 10.31662/jmaj.2024-0043 (PMC11301014; doi:10.31662/jmaj.2024-0043)
Supplement: Supplementary Table 1 [file 2433-3298-7-3-0353-s003.pdf]

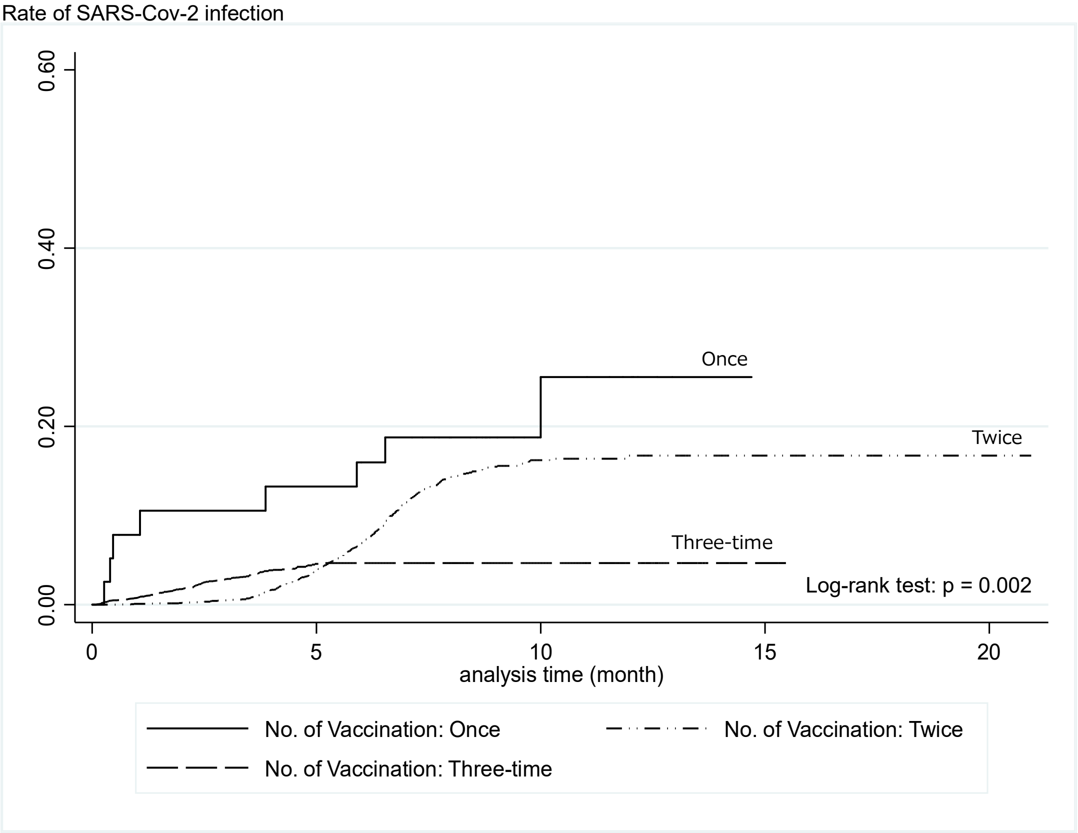

[Cox proportional hazards model]

The number of vaccinations: HR = 0.75 (95%CI: 0.62-0.91)

* Adjusted by age (categorical), underlying health condition, exercise habit, smoking habit (categorical) and drinking habit (categorical), sleep status, BMI (categorical), and breakfast consumption

Supplementary Figure 1 Time dependent variables of the final date of vaccination and SARS-CoV-2 infection (The first period: 2021.4-2022.5)
