# Supplementary material for: How Healthy Lifestyle Habits Have Interacted with SARS-CoV-2 Infection and the Effectiveness of COVID-19 Vaccinations: Tohoku Medical Megabank Project Birth and Three-Generation Cohort Study
Source: JMA J. 2024 Jul 3;7(3):353–63. doi: 10.31662/jmaj.2024-0043 (PMC11301014; doi:10.31662/jmaj.2024-0043)
Supplement: Supplementary Table 2 [file 2433-3298-7-3-0353-s004.pdf]

	Exercise habit		Smoking habit			Drinking habit				Sleep status		BMI				Breakfast consumption	
	Having	Non	Non	Past	Current	Non	Modeate	More than moderate	Heavy	Satisfaction	Dissatisfaction	Underweight	Standard	Overweight	Obesity	Everyday	Others
	OR (95%CI)	OR (95%CI)	OR (95%CI)	OR (95%CI)	OR (95%CI)	OR (95%CI)	OR (95%CI)	OR (95%CI)	OR (95%CI)	OR (95%CI)	OR (95%CI)	OR (95%CI)	OR (95%CI)	OR (95%CI)	OR (95%CI)	OR (95%CI)	OR (95%CI)
**[Pre-vaccination phase]** 2020.3-2021.3	0.74 (0.31-1.79)	1.00	0.41 (0.15-1.08)	0.59 (0.21-1.64)	1.00	0.46 (0.18-1.19)	0.28 (0.06-1.31)	0.40 (0.11-1.39)	1.00	0.67 (0.32-1.42)	1.00	0.87 (0.26-2.95)	1.00	**3.07 (1.45-6.49)**	-	0.63 (0.29-1.39)	1.00
**[First period]** 2021.4-2022.5	0.91 (0.76-1.10)	1.00	0.90 (0.71-1.15)	1.10 (0.86-1.41)	1.00	1.08 (0.86-1.37)	1.05 (0.80-1.39)	0.88 (0.66-1.18)	1.00	0.90 (0.77-1.05)	1.00	0.91 (0.72-1.15)	1.00	0.92 (0.73-1.15)	1.18 (0.79-1.77)	0.97 (0.82-1.16)	1.00
**[Second period]** 2022.6-2023.5	1.08 (0.96-1.21)	1.00	1.08 (0.92-1.27)	1.12 (0.95-1.33)	1.00	1.03 (0.89-1.21)	1.01 (0.84-1.21)	1.07 (0.89-1.28)	1.00	0.99 (0.90-1.10)	1.00	0.91 (0.78-1.07)	1.00	1.07 (0.93-1.23)	0.93 (0.69-1.24)	1.04 (0.92-1.17)	1.00

* OR: Odds ratio, Logistic regression analysis (adjusted by age (categorical), sex and underlying health condition) Bold : p <0.05

Odds ratio : 0.00-0.25 | 0.25-0.50 | 0.50-0.75 | 0.75-1.00 | 1.00-1.25 | 1.25-1.50 | 1.50-

Supplementary Figure 2 Visualization of interaction between lifestyle habits and the number of vaccinations against SARS-CoV-2 infection (regardless of the COVID-19 vaccination)
